# Direct Growth of Graphene on Insulator Using Liquid Precursor Via an Intermediate Nanostructured State Carbon Nanotube

**DOI:** 10.1186/s11671-019-2935-9

**Published:** 2019-03-22

**Authors:** Pramoda K. Nayak

**Affiliations:** Department of Physics, Indian Institute of Technology Madras, Chennai, 600036 India

**Keywords:** Graphene, Carbon nanotube, Ethanol, Chemical vapor deposition

## Abstract

Synthesis of high-quality graphene layers on insulating substrates is highly desirable for future graphene-based high-speed electronics. Besides the use of gaseous hydrocarbon sources, solid and liquid hydrocarbon sources have recently shown great promises for high-quality graphene growth. Here, I report chemical vapor deposition growth of mono- to few-layer graphene directly on SiO_2_ substrate using ethanol as liquid hydrocarbon feedstock. The growth process of graphene has been systematically investigated as a function of annealing temperature as well as different seed layers. Interestingly, it was found that the carbon atoms produced by thermal decomposition of ethanol form sp^2^ carbon network on SiO_2_ surface thereby forming nanographene flakes via an intermediate carbon-based nanostructured state carbon nanotube. This work might pave the way to an understanding for economical and catalyst-free graphene growth compatible with current silicon-processing techniques, and it can be applied on a variety of insulating surfaces including quartz, sapphire, and fused silica.

**Electronic supplementary material:**

The online version of this article (10.1186/s11671-019-2935-9) contains supplementary material, which is available to authorized users.

## Introduction

Synthesis of high-quality graphene on insulators is highly desirable toward the development of graphene-based electronic devices to avoid the deleterious metallic effects caused by conventional metal catalytic assisted graphene growth using chemical vapor deposition (CVD) [1–4]. One of the potential methods to achieve graphene growth on insulator is by introducing a metal catalyst in the vapor phase, which has been recently demonstrated by Teng et al. [5] and Kim et al. [6]. The catalyst metal in a vapor phase would react with carbon precursor in the gas phase as well as on the surface of the insulating substrate, leading to high-quality uniform graphene formation. Another way is to grow graphene directly on dielectric insulators without using any metal catalyst, which is a much-needed technique for its electronic application. Recently, many research groups have pursued the direct growth of graphene on various dielectric substrates including hexagonal boron nitride (h-BN) [7, 8], glass [9–11], quartz [12], sapphire [13, 14], Si_3_N_4_ [15–17], SiO_2_ [18–21], and high-k dielectrics such as MgO [22, 23], ZrO_2_ [23], and TiO_2_ [24], using CVD without using metal catalysts. However, the as-grown graphene on above substrates exhibits poor quality, which is comparable neither with that of graphene grown on metal substrates such as Ni [1] and Cu [3] nor with epitaxial graphene on SiC [25]. Moreover, the graphene growth mechanism of the above systems is also not well understood.

Besides gaseous precursor, the growth of graphene using wider variety of potential feedstocks such as solid and liquid hydrocarbon is highly in demand in order to meet its technological application. Since the last 5 years, many groups have synthesized graphene using solid and liquid hydrocarbon feedstock other than methane using the revised CVD route [26–29]. In the above works, metal catalysts were used for graphene nucleation. Moreover, the synthesis of high-quality graphene using the above hydrocarbon feedstocks requires a deep understanding of the growth mechanism. Recently, monolayer graphene on Cu using a liquid hydrocarbon, i.e., ethanol, has been demonstrated by Zhao et al. [30], where growth mechanism is reported to be self-limiting. The idea behind choosing ethanol as carbon source lies on its following advantages including environmental friendly, comparatively cheaper, easier to use, and less flammable than high purity methane, thus making graphene fabrication more accessible [28]. Using ethanol as the carbon source, high-quality monolayer graphene with an *I*_D_/*I*_G_ of ∼ 0.04 at a lower reaction temperature of ~ 800 °C was obtained by Zhao et al. [30], which indicates that ethanol outstretches methane in CVD synthesis of graphene on Cu foil. Although many groups have reported growth mechanism of graphene on insulators using methane [13, 31], and graphene on metal substrates using solid and liquid hydrocarbon feedstock [26, 27, 30], but a comprehensive growth mechanism of graphene directly on insulator using liquid hydrocarbon feedstock is lacking in the state-of-the-art research and requires further exploration.

In the present work, I propose a novel growth technique that enables direct formation of mono- to few-layer graphene on SiO_2_ using ethanol as a carbon precursor in CVD, and systematically investigate its growth process as a function of annealing temperature and different seed layers. The prime feature of the growth mechanism includes the following steps: (1) decomposition of liquid hydrocarbon in a gas phase; (2) graphitization of carbon atoms on silicon oxide surfaces to form intermediate phases including carbon nanoclusters and carbon nanotubes (CNTs); (3) etching by hydrogen at elevated temperature that leads to formation of graphitic nanoribbon, which acts as nucleation sites for graphene growth; and (4) combination of these graphitic nanoribbons to form continuous high-quality graphene films after prolonged annealing time.

## Methods

### Growth of Graphene on SiO_2_

Graphene growth on silica was carried out by atmospheric pressure chemical vapor deposition (APCVD) by using liquid hydrocarbon feedstock ethanol as carbon source. Prior to growth, 300-nm wafer scale SiO_2_/Si substrates were cleaned by acetone and isopropyl alcohol (IPA) with sonication, followed by N_2_ gas purging. These substrates were placed in the upstream gas flow and heated up to 1100 °C with a heating rate of 10 °C/min in the presence of H_2_ (40 sccm) and Ar (250 sccm) at ambient pressure. At this temperature, substrates were held for 5–10 min to maintain temperature stability followed by graphene growth stage of 5 min. During this growth stage, carrier gas Ar (10 sccm) was passed through a U-shaped quartz tube containing ethanol to carry this hydrocarbon vapor into the horizontal quartz tube (reaction zone) in CVD as shown in Additional file 1: Figure S1. This experiment was repeated for 10-, 15-, and 60-min growth respectively, followed by cooling to room temperature to obtain continuous graphene film on SiO_2_ substrates.

For seed layer-assisted graphene growth, the SiO_2_ substrates were covered with different seed materials such as exfoliated graphene, charcoal, electron cyclotron resonance (ECR) graphene, and CVD graphene prior to growth and carrier gas Ar (4 sccm) was supplied at 1100 °C for 1-h growth time. Following growth, the samples were characterized by Raman spectroscopy, which reveals the characteristic features of as-grown carbon nanostructured films including CNT and graphene and also allows for the identification of single- and multiple-layer graphene. Other characterization methods including transmission electron microscopy (TEM), scanning electron microscopy (SEM), and X-ray photoelectron spectroscopy (XPS) were employed to study crystallinity, surface morphology, and compositional analysis of as-prepared graphene layers.

### Growth of ECR-CVD Graphene for Seed Layer

Commercially available 300-nm SiO_2_/Si substrate was first cleaned in acetone, isopropyl alcohol, and de-ionized water. After cleaning, the substrate was placed in the ECR-CVD chamber. The schematic of the ECR-CVD chamber is shown in Additional file 1: Figure S2. When the vacuum reached 1 × 10^−6^ Torr, Ar flow was introduced at a rate of 5 sccm, and the plasma was ignited at a partial pressure of 6 × 10^−3^ Torr at 400 W for 5 min to remove organic residues from the substrate surface. The temperature was then raised to 600 °C under high vacuum. When the temperature stabilized, argon and ethylene flows (Ar:C_2_H_4_ = 0.3:0.15 sccm) were opened for 30 s and plasma power was set at 1600 W, followed by annealing in 1 sccm H_2_ flow for 5 min at the same temperature. Finally, the sample was cooled down to room temperature under high vacuum.

## Results and Discussion

### CVD Growth of Graphene Using Ethanol

**Fig. 1:**
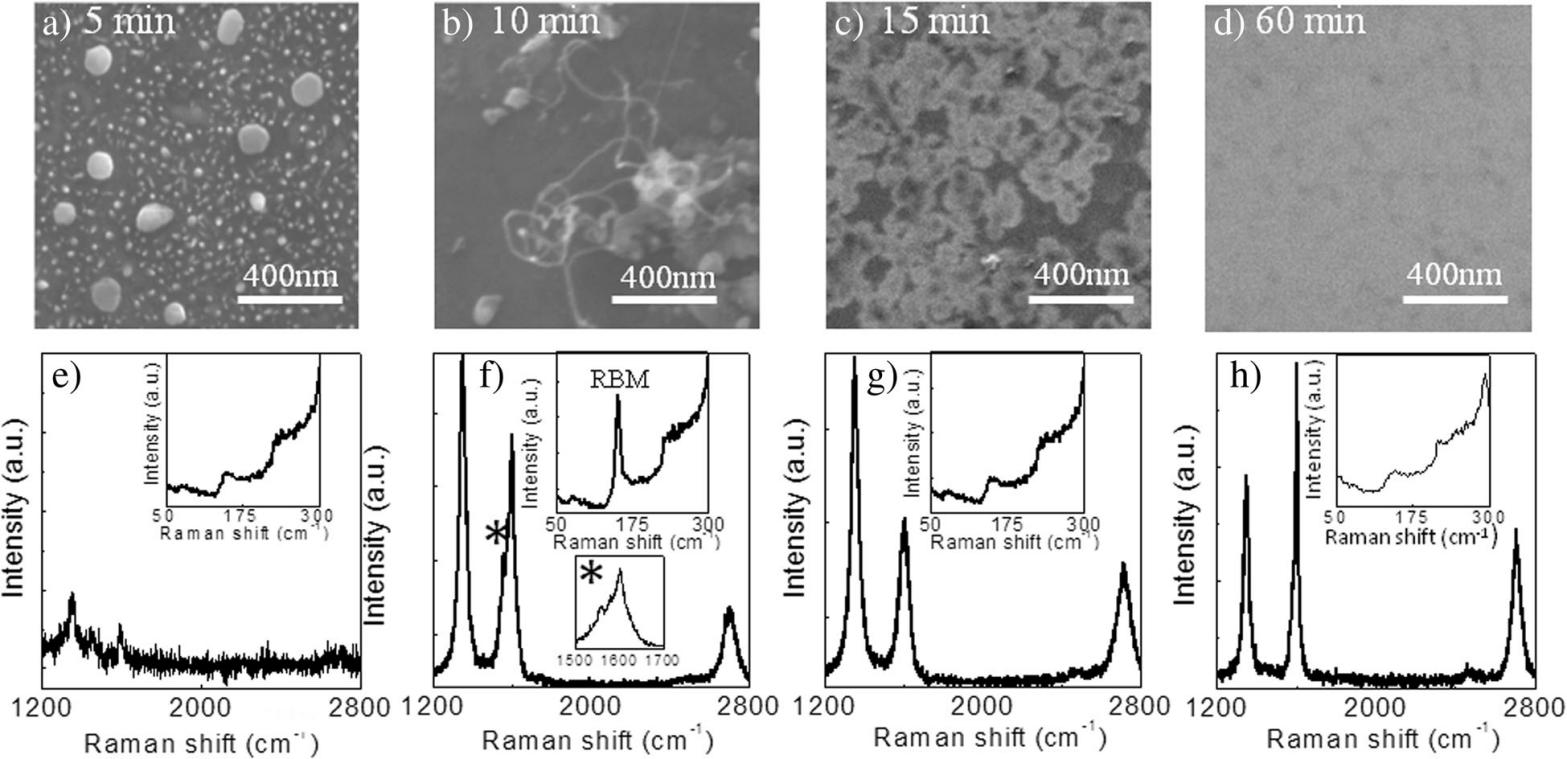
SEM images of carbon-based nanostructures directly grown on silica at 1100 °C for growth time of **a** 5 min, **b** 10 min, **c** 15 min, and **d** 60 min. **e**–**h** Their respective Raman spectra in the frequency range 1200 to 2800 cm^−1^. The Raman features in the frequency range 50–300 cm^−1^ are illustrated as insets in **e**–**h**. G band splitting in **f** around 1560 cm^−1^ marked as asterisk and the presence of the RBM peak near 150 cm^−1^ indicates the formation of SWCNT

Ethanol was used as a carbon source for growth of graphene on SiO_2_ substrate using ethanol vapor-based CVD system as shown in Additional file 1: Figure S1. In brief, graphene growth was carried out at 1100 °C in the presence of H_2_ and Ar with a flow rate of 40 sccm and 250 sccm respectively, in ambient pressure. Figure 1a–d shows the SEM images of various carbon nanostructures grown on SiO_2_ substrate for different growth time in the range of 5–60 min, and their representative micro Raman spectra are depicted in Fig. 1e–h. For 5-min growth duration, it is observed that carbon nanoparticles are formed, represented by big and small white circles (Fig. 1a). These nanoparticles are amorphous in nature as confirmed from the Raman spectrum (Fig. 1e) [32]. The inset shows the Raman feature in the frequency range of 50–300 cm^−1^. When the growth time extends to 10 min, some of the carbon nanoparticles transform into CNTs as can be seen in Fig. 1b. The Raman G peak splitting occurs at around 1560 cm^−1^ (Fig. 1f) marked as asterisk, which is due to the spirally feature of C-network and presumed to be characteristics of CNTs [33, 34]. Further, observation of strong radial breathing mode (RBM) peak close to 150 cm^−1^ confirms the formation of single-wall carbon nanotube (SWCNTs) [35, 36]. After 15-min growth, there is a complete transformation of CNTs to graphene that appeared with some defect structures as confirmed from strong D peak intensity (Fig. 1g). The white areas in the SEM image correspond to mono- to few-layer graphene, where the black areas are the substrate. When the growth time further extended to 60 min, complete coverage of graphene was observed from the SEM image with fewer defects (Fig. 1d). In addition, Raman spectra also confirmed the formation of graphene with a relatively low defect resulting from reduced D peak intensity (Fig. 1h).

**Fig. 2:**
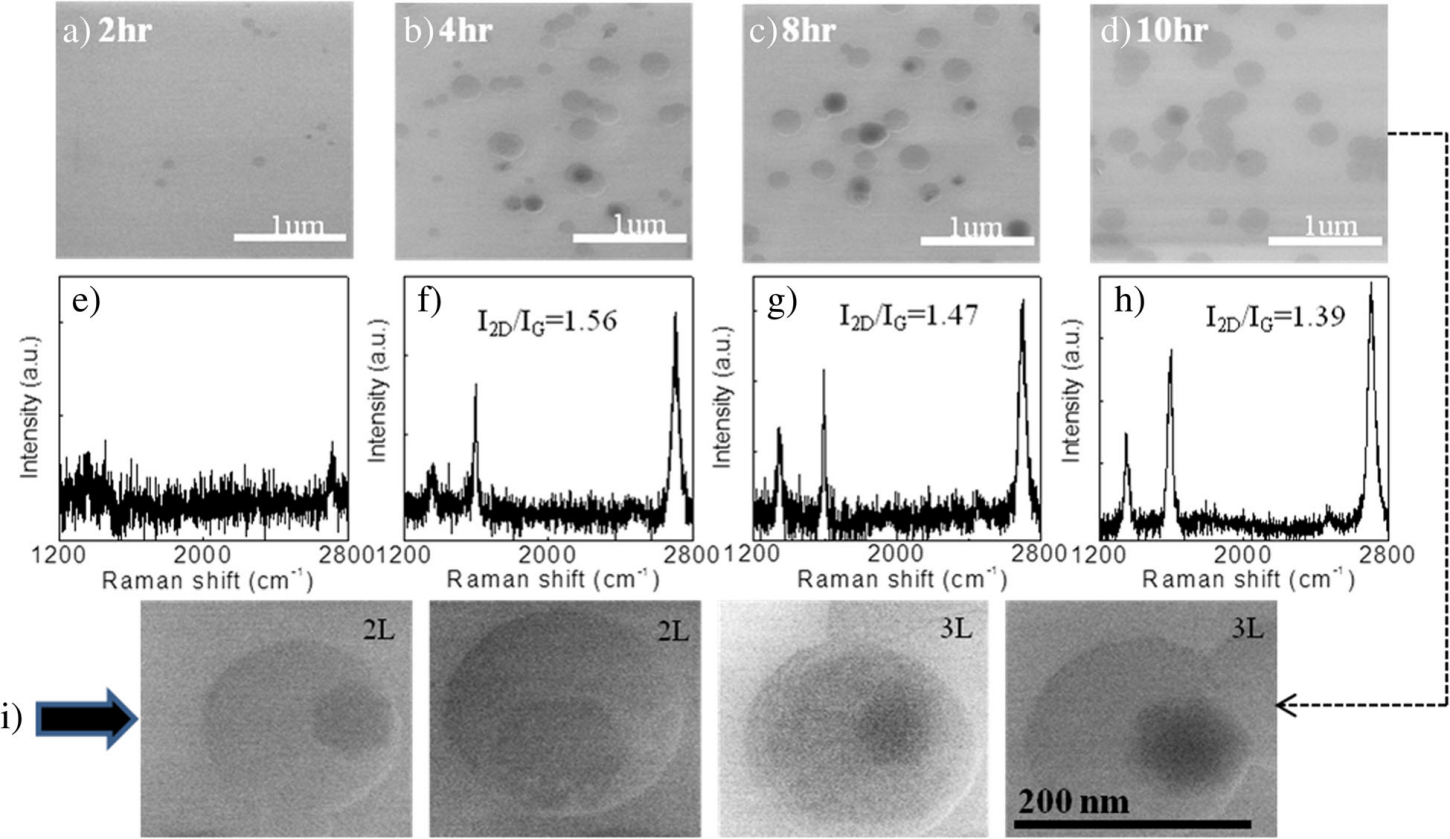
SEM images of the graphene directly grown on silica at 1150 °C for growth time of **a** 2 h, **b** 4 h, **c** 8 h, and **d** 10 h. Their representative Raman spectra are shown in **e**–**h**. The *I*_2D_/*I*_G_ ratio for 4-h-, 8-h-, and 10-h-grown graphene is estimated to be 1.56, 1.47, and 1.39, respectively. Bottom panel **i** presents some high-magnification SEM images of bilayer and trilayer graphene flakes taken from **d**. The grains with larger in size and lower contrast are first graphene layers, and those with smaller in size and higher contrast correspond to the second and third layers. The scale bar is the same for all

Furthermore, growth was carried out at a higher growth temperature of 1150 °C using reduced carrier gas (Ar) flow of 3 sccm. Figure 2a–d displays the SEM images of the graphene grown for different growth time in the range from 2 to 10 h, and their representative Raman spectra are depicted in Fig. 2e–h. For short growth time (2 h), the substrate is covered with very few graphene flakes observed from both SEM and Raman data. When growth period extends to 4 h, 8 h, and 10 h, the density of graphene flakes increases and the substrate is filled with mostly monolayer coverage (flakes with white contrast) and few bilayer coverage (flakes with both white and black contrast) as confirmed from their Raman analysis (*I*_2D_/*I*_G_ ~ 1.5). But there is no systematic change in *I*_2D_/*I*_G_ ratio as well as the density of flakes observed beyond 4-h growth time. The bottom panel Fig. 2i illustrates some high-magnification SEM images of graphene flakes taken from Fig. 2d, where hexagonal-shaped graphene flakes are completely visible in bilayer and trilayer regions. The above observation indicates that longer growth time enhances the graphene nucleation density followed by saturation after a certain threshold time duration, which implies that the seed sites for nucleation are few and achieve saturation after initial growth. Although the yield of graphene growth using this technique is ~ 80% which is very less than that of CVD graphene grown using gaseous precursor (~ 95%) [3], but it has an advantage in terms of catalyst-free graphene growth directly on an insulating substrate which avoids the need of complicated post growth transfer process.

**Fig. 3:**
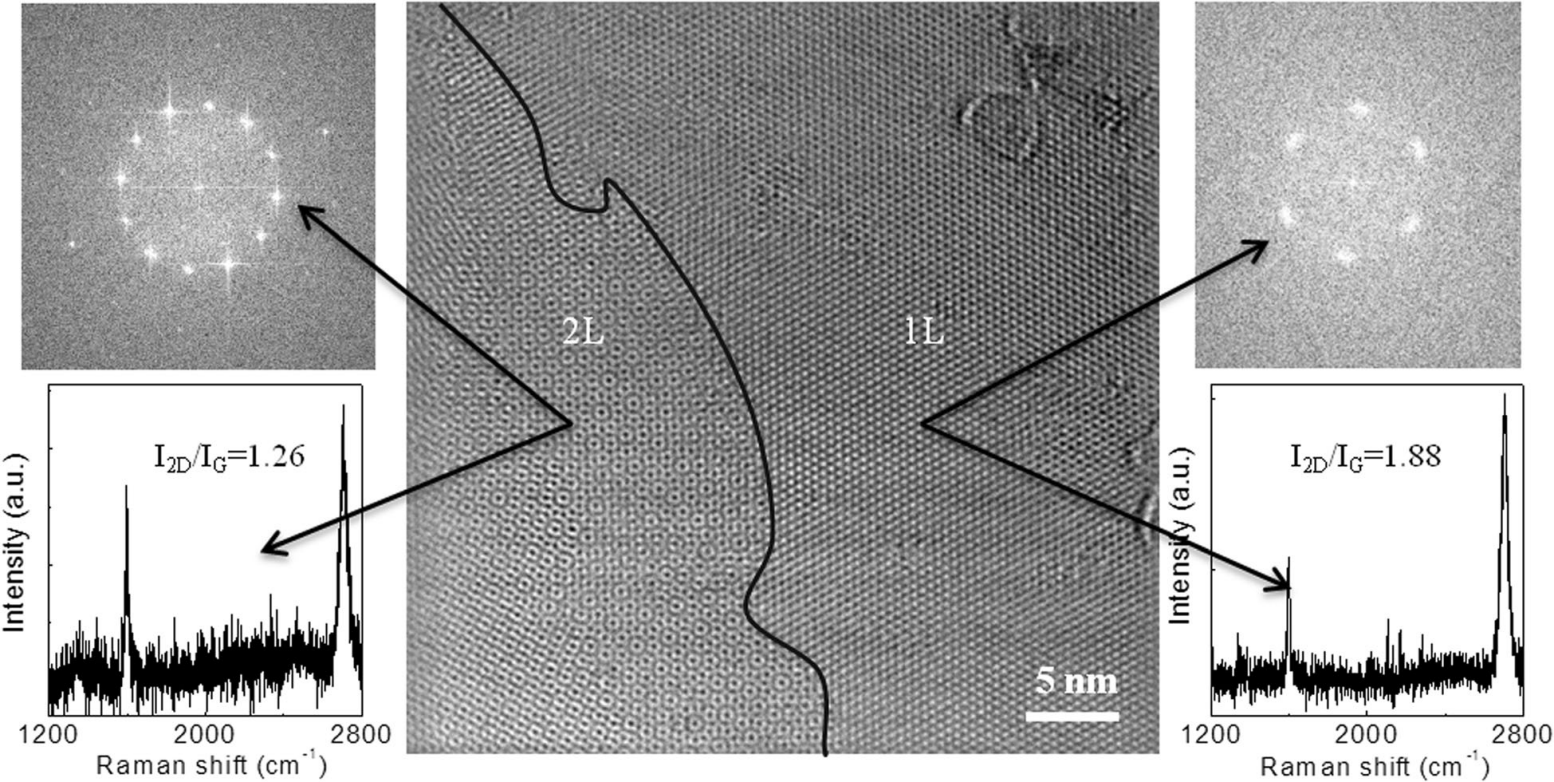
TEM analysis of a CVD-grown graphene flake from ethanol prepared with 4-h growth time, taken from Fig. 2b. High-resolution TEM image showing both monolayer and bilayer regions separated by marked line. Monolayer region contains some defects, which is localized. Moiré patterns as a result of rotational misalignment of the two graphene layers are clearly seen in the bilayer region. The right and left panels of the TEM image display the Fourier transform of the electron diffraction patterns of 1L and 2L graphene. Hexagonal selected area electron diffraction patterns of the monolayer and bilayer graphene reveal the nice crystallinity. Their representative Raman spectra are illustrated in the bottom panel with *I*_2D_/*I*_G_ ratio of 1.88 and 1.26 for 1L and 2L, respectively

Figure 3 reveals the low-magnification TEM image of a graphene flake, prepared at 1150 °C for 4 h (Fig. 2b), which consists of both single-layer and bilayer regions with some defects. The single-layer graphene (shown in the right part) consists of hexagonal carbon lattices, which could be seen from the Fourier transform of the electron diffraction pattern (right panel) with large *I*_2D_/*I*_G_ ratio (1.88) observed from the Raman spectrum. The left part of the TEM image contains Moiré patterns as a result of rotational misalignment of the two graphene layers [37]. Furthermore, the Fourier transform and *I*_2D_/*I*_G_ ratio (~ 1.26) observed from the Raman spectrum (left panel) illustrate the bilayer feature of the graphene layers.

### Graphene Growth Mechanism on SiO_2_

**Fig. 4:**
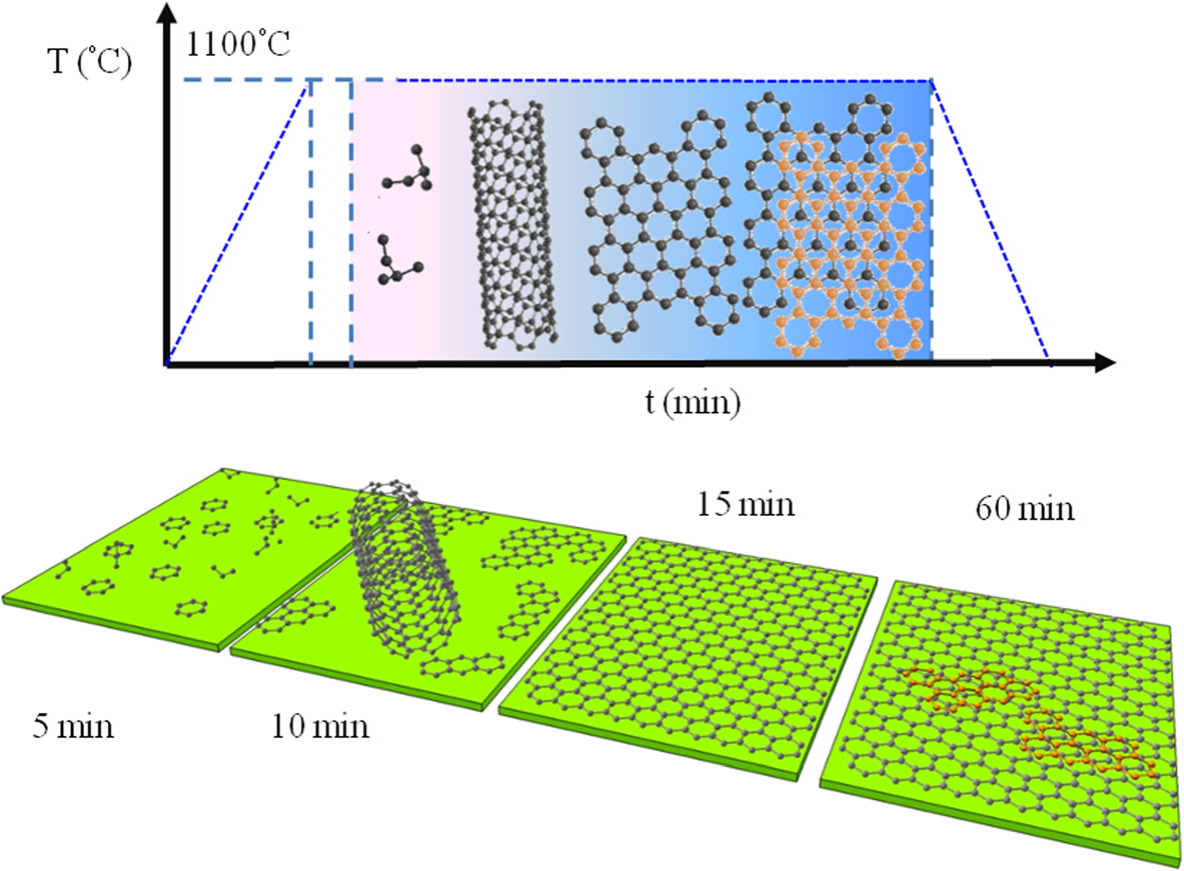
Schematic illustration of graphene growth mechanism on SiO_2_ from ethanol. Bottom panel depicts the evolution of carbon-based nanostructures including carbon nanoparticles, mixture of CNTs and carbon nanoparticles, multilayer graphene (MLG) with some defects, and mono- to few-layer graphene for growth time of 5, 10, 15, and 60 min, respectively

Based on the above observation, I propose the following graphene growth mechanism from ethanol as illustrated in Fig. 4. Different carbon-based nanostructures such as carbon nanoparticles, mixture of CNTs and carbon nanoparticles, multilayer graphene (MLG) with defects, and mono- to few-layer graphene are found to be evolved for growth time of 5, 10, 15, and 60 min, respectively. Step 1 begins with the thermal decomposition of ethanol vapor at ambient pressure and elevated temperature (~ 700 °C), which energetically favors the formation of ethylene gas and water as per the following reaction [38]:

1 $$ {\mathrm{C}}_2{\mathrm{H}}_5{\mathrm{OH}}^{\left(\mathrm{g}\right)}\to {\mathrm{C}}_2{{\mathrm{H}}_4}^{\left(\mathrm{g}\right)}+{\mathrm{H}}_2\mathrm{O} $$

H_2_ gas helps in the further decomposition of ethylene into carbon and hydrogen atoms. Relatively large adsorption energy of liquid precursor compared to gaseous precursor suggests that trapping-mediated growth process is more relevant here [27]. Moreover, the step edges of the SiO_2_ substrate (see Additional file 1: Figure S3) can potentially act as defect sites in which carbon atoms get trapped onto it and start nucleate, as already discussed in the case of transition metal dichalcogenides growth [39]. For short growth time (5 min), the decomposition of ethylene to carbon may not be completed, and disordered hydrocarbon structures would therefore develop on the substrate. When growth time extends to 10 min, decomposition of the above molecule comes closer to completion. In this case, some of the carbon atoms arrange orderly in a spiral configuration to form CNTs and some remain in amorphous. It can be noted that CNT growth is mostly preferred from the decomposition of ethanol at an elevated temperature with proper hydrogen flow [40, 41].

For 15-min growth time, the decomposition perhaps complete and the carbon atoms connect to each other in plane to form C-C sp^2^ network called graphene islands. Further increase in growth time to 60 min accounts for the expansion of graphene islands and forms graphene layers. Above growth process suggests that direct CVD growth of graphene on insulators require high growth temperature and long deposition time as compared to those on metallic substrates [42]. It is predicted that both H_2_ gas and H_2_O remove the sp^3^ network as well as amorphous carbon atoms present there and only retain sp^2^ network for prolonged growth time. This growth mechanism is similar to the oxygen-aided synthesis of polycrystalline graphene on silicon dioxide substrates [18].

### Seed-Assisted Growth of Graphene

**Fig. 5:**
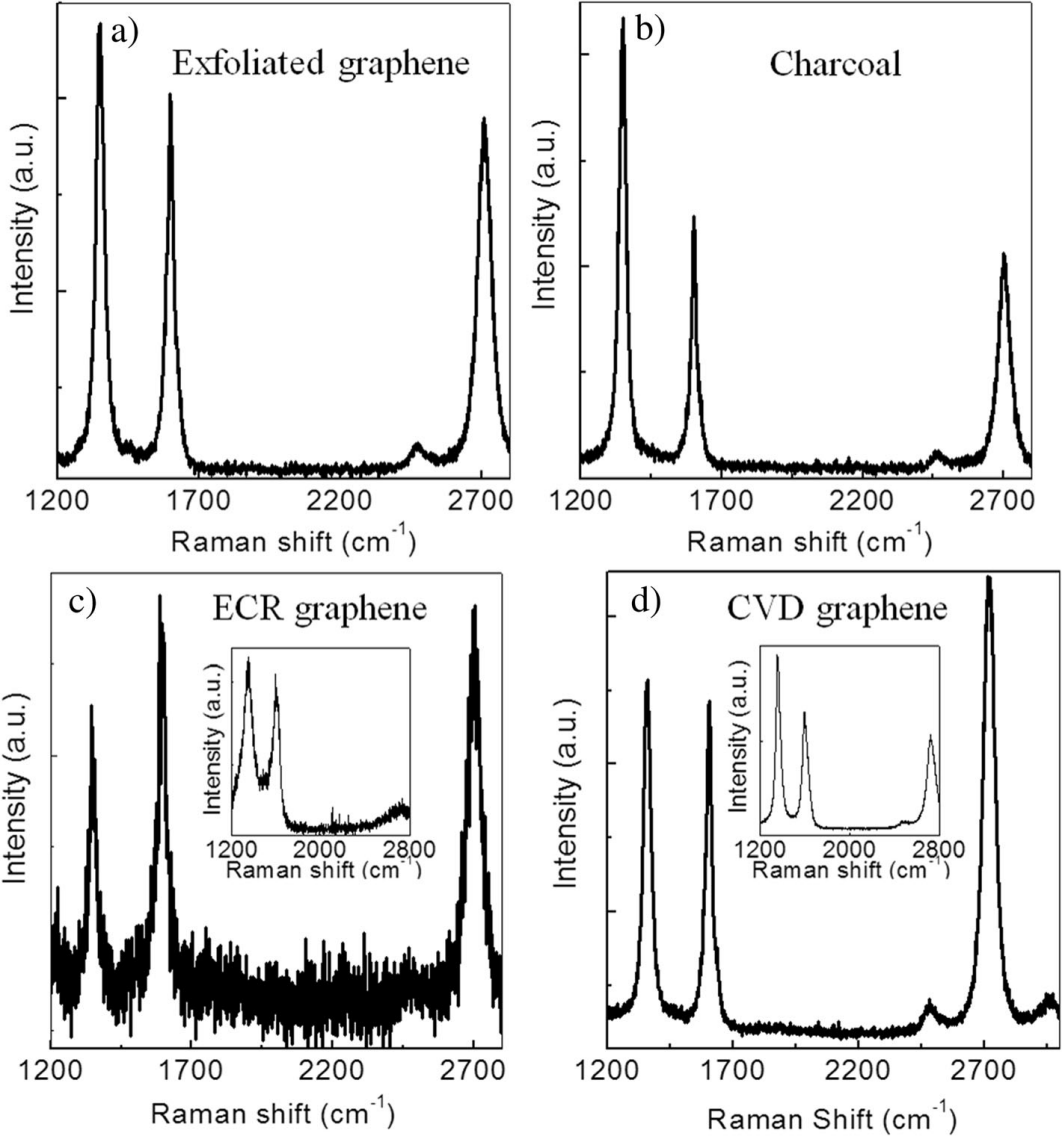
Raman spectra of graphene grown by CVD for different seed materials including **a** mechanically exfoliated graphene, **b** charcoal, **c** ECR-CVD graphene, and **d** CVD graphene. The insets in Fig. 5c and d display the representative Raman spectrum of graphene seeds prepared by ECR-CVD and CVD route, respectively

Seed-assisted growth of graphene has already been demonstrated by several groups [43, 44], to control surface nucleation and to optimize the quality of graphene films. The nucleation of CVD graphene on Cu has been able to control using polymethyl methacrylate (PMMA) as pre-patterned seeds [44]. But while preparing graphene directly on insulator, there still remain great opportunities to explore the graphene growth and its associated mechanism using different seed materials as nucleation sites. By considering the above aspects, four seed materials including exfoliated graphene, charcoal, ECR-CVD graphene, and CVD graphene were chosen in the present study to explore the graphene growth as illustrated in Fig. 5.

When mechanically exfoliated graphene was used as seed on SiO_2_ substrate, the Raman spectrum (Fig. 5a) reveals the characteristics of two- to three-layer graphene along with large D-band intensity, which indicates that prepared graphene has high defect density. This is unlikely with the previous report [5], where the graphene film exhibited very low defect density by using the same seed material in CVD synthesis. Cu vapor acted as the catalyst in that case, which controlled the nucleation rate, thereby resulting in high-quality graphene. But in the present case, the rate of nucleation is not controlled, which results in graphene with high defect density. Figure 5b displays the Raman spectrum of graphene by taking charcoal as seed material. The feature of the resulting graphene is similar to the previous one (Fig. 5a) with large defect density.

Then, I have chosen graphene prepared by ECR-CVD method [45] as the seed material for graphene growth (Fig. 5c). Compared to mechanically exfoliated graphene and charcoal seed-based graphene, ECR-CVD seed-based graphene exhibits reduced D-band intensity, indicating low defect density. But it is worth noting that the Raman spectrum shows a large noise level which is related to the degree of cleanness like in Fig. 2. It can also be due to incomplete growth or partial growth leading to lower signal. ECR-CVD growth of nanographene was carried out at 600 °C in the presence of C_2_H_2_ and Ar with the flow rate (C_2_H_2_:Ar = 0.15:0.3 sccm) for 30 s and plasma power of 1600 W, followed by annealing in 1 sccm H_2_ flow for 5 min. The distance between substrate and plasma was kept to be 5 cm. The detailed synthesis of the ECR-CVD nanographene has been mentioned in the experimental section, and its Raman feature is displayed as an inset in Fig. 5c. Finally, CVD graphene was chosen as the seed for graphene growth, and its Raman spectrum is displayed in Fig. 5d. Almost single-layer graphene is formed with large symmetric Gaussian 2D peak intensity (*I*_2D_/*I*_G_ ~ 1.35), which indicates high-quality graphene. The graphene seed was synthesized at 1100 °C for 1-h growth time in the presence of Ar and H_2_ flow (Ar:H_2_ = 250:40 sccm) with carrier gas Ar flow of 4 sccm, and its Raman spectrum is shown as inset in Fig. 5d.

XPS was used to investigate the elemental analysis of the prepared graphene in this work. Additional file 1: Figure S4 shows the XPS spectra of the graphene film grown directly on SiO_2_ at 1100 °C for 1 h. No other peaks are found except Si2s, Si2p, O1s, and O2s, which are the contribution from SiO_2_. The bottom figure depicts the C1s core level spectrum. The only narrow and symmetric intense peak at 284.4 eV with a full width half maximum (FWHM) of 1.91 eV is assigned to the sp^2^-bonded C atom, signifying the formation of nanographene using this metal-free method, similar to the previous work [5].

### Graphene Growth as a Function of Growth Temperature

**Fig. 6:**
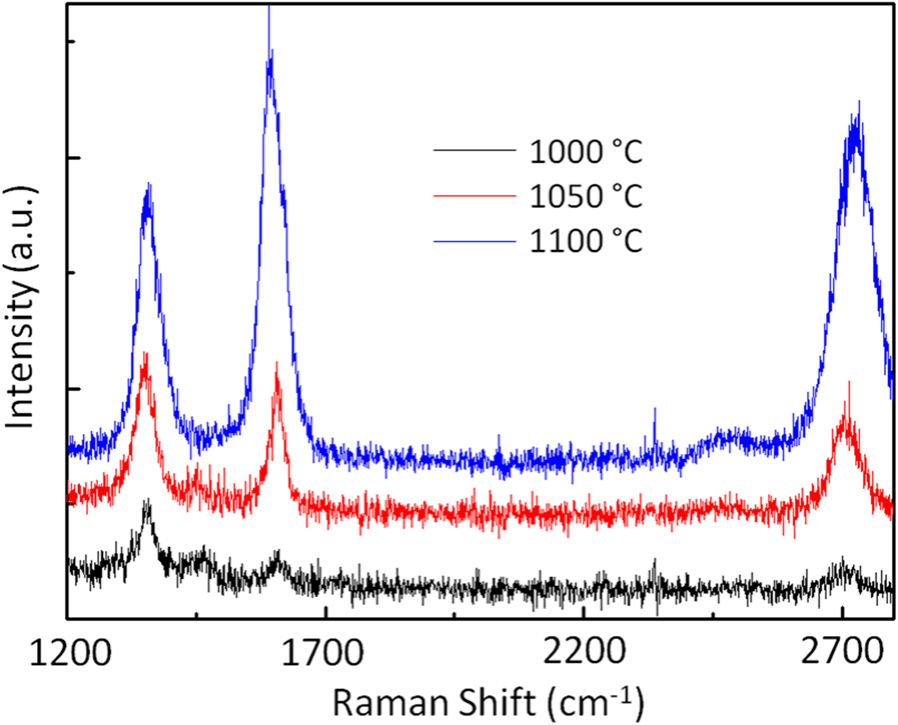
Micro-Raman characterization of CVD graphene grown directly on SiO_2_ for different growth temperatures. 1000 °C (black), 1050 °C (red) 1100 °C (blue)

I have systematically investigated the CVD growth of graphene on SiO_2_ as a function of growth temperature by keeping other parameters fixed including diluted gas flow rate (Ar:H_2_ = 235:40 sccm) and carrier gas flow rate (Ar = 10 sccm). Three growth temperatures (1000 °C, 1050 °C, and 1100 °C) were selected, and their representative Raman spectra are displayed in Fig. 6. For low growth temperatures, 1000 °C and 1050 °C, broad 2D peak with intensity less than G peak was observed, indicating the formation of multiple graphene layers due to uncontrolled random nucleation on the bare oxide surface. Furthermore, the presence of large D peak intensity indicates the signature of high-defect density graphene. The random nucleation and poor surface migration of carbon atoms are presumably the major causes of defects in this growth process. When the growth temperature exceeded to 1100 °C, the quality of graphene appeared to be good with relatively large and sharp 2D peak intensity, but the D peak still exists there which depicts finite defect density. It is expected that high growth temperature promotes some controlled nucleation, which is responsible for improved graphene quality. Hence, 1100 °C was assumed to be the optimum temperature for synthesizing high-quality graphene directly on SiO_2_ in CVD.

My proposed graphene growth mechanism on insulator is based on the thermal decomposition of ethanol, followed by controlled nucleation of carbon 2D islands to form continuous nanographene via an intermediate state CNT. In order to further validate the above hypothesis, CNT was taken as the source material for graphene in CVD and its transformation was studied as a function of growth time as illustrated in Raman mapping (Additional file 1: Figure S5). First, CNT was placed on SiO_2_ substrate before CVD growth as shown as schematic in the top panel figure. After 5-min growth, there is an appearance of distorted CNT structure, and finally, it transforms to complete graphene, when growth time approaches 10 min. The RBM, 2D/G, and D/G Raman mapping of 20 × 20 μm^2^ region are displayed in the bottom panel figure, revealing the evolution of graphene structure as a function of growth time. The RBM peak assigned to white rectangles in the Raman mapping is the signature of CNT that is present initially and vanishes after 10-min growth time. 2D/G peak (white rectangles) is low for CNT and increases with increasing growth time. Similarly, the D/G peak (black rectangles), which is the signature of defects, is high for CNT and reduces drastically for graphene (10-min growth time).

## Conclusions

In summary, I have demonstrated a novel graphene growth technique directly on silicon oxides in chemical vapor deposition using ethanol as carbon precursor other than methane. Decomposition of ethanol to ethylene and water followed by nucleation of sp^2^ network carbon on SiO_2_ surface, which acts as nucleation center, leads to the formation of nanographene flakes via an intermediate nanostructured carbon state CNT. The growth of graphene has been systematically investigated as a function of annealing temperature and seed layer, and finally, its self-limiting behavior has been discussed. It is observed that higher growth temperature and lower carrier gas flow enhance the crystalline quality of graphene flakes. CVD graphene is promised to be the best seed layer compared to exfoliated graphene, charcoal, and ECR graphene to obtain high quality of graphene flakes. The proposed method avoids the need for either a metal catalyst or a complicated and skilled post growth transfer process and paves a way toward the development of practical applications for graphene, especially in electronics requiring integration with current Si processing technology.

## Additional file

Additional file 1: **Figure S1.** Schematic of the CVD growth of graphene from ethanol on SiO_2_/Si substrate. **Figure S2.** Schematic of ECR-CVD chamber. **Figure S3.** AFM image of 300 nm SiO_2_Si substrate showing step edges and surface roughness around 300 pm. **Figure S4.** a. XPS spectrum of the graphene film grown directly on SiO_2_ at 1100 °C for 1 h. b. Evolution of C1s core-level spectrum of graphene grown on SiO_2_ for the same parameters. The narrow and symmetric intense peak at 284.4 eV assigned to the sp^2^-bonded C-network. **Figure S5.** a. Schematics of graphene growth on SiO_2_ using CNT as source material. b. Raman intensity maps for RBM, 2D/G ratio, and D/G ratio in 20 × 20 μm^2^ regions of carbon nanostructures formed at different growth time 0, 5, and 10 min. The white rectangles in the Raman mapping correspond to RBM peak of CNT, which vanishes after 10-min growth time. The density of white rectangles (2D/G peak) is low for CNT and increases with increasing growth time. The density of black rectangles (D/G peak), which is the signature of defects is high for CNT and reduces drastically for graphene (10-min growth time) (DOCX 2248 kb)

## Abbreviations

AFM: Atomic force microscopy
CNTs: Carbon nanotubes
CVD: Chemical vapor deposition
ECR-CVD: Electron cyclotron resonance-CVD
FWHM: Full width half maximum
MLG: Multilayer graphene
PMMA: Polymethyl methacrylate
RBM: Radial breathing mode
SEM: Scanning electron microscopy
SWCNT: Single-wall carbon nanotube
TEM: Transmission electron microscopy
XPS: X-ray photoelectron spectroscopy
