# Supplementary material for: Direct Growth of Graphene on Insulator Using Liquid Precursor Via an Intermediate Nanostructured State Carbon Nanotube
Source: Nanoscale Res Lett. 2019 Mar 22;14:107. doi: 10.1186/s11671-019-2935-9 (PMC6430270; doi:10.1186/s11671-019-2935-9)
Supplement: Supplementary file 1 — Figure S1. Schematic of the CVD growth of graphene from ethanol on SiO2/Si substrate. Figure S2. Schematic of ECR-CVD chamber. Figure S3. AFM image of 300 nm SiO2Si substrate showing step edges and surface roughness around 300 pm. Figure S4. a. XPS spectrum of the graphene film grown directly on SiO2 at 1100 °C for 1 h. b. Evolution of C1s core-level spectrum of graphene grown on SiO2 for the same parameters. The narrow and symmetric intense peak at 284.4 eV assigned to the sp2-bonded C-network. Figure S5. a. Schematics of graphene growth on SiO2 using CNT as source material. b. Raman intensity maps for RBM, 2D/G ratio, and D/G ratio in 20 × 20 μm2 regions of carbon nanostructures formed at different growth time 0, 5, and 10 min. The white rectangles in the Raman mapping correspond to RBM peak of CNT, which vanishes after 10-min growth time. The density of white rectangles (2D/G peak) is low for CNT and increases with increasing growth time. The density of black rectangles (D/G peak), which is the signature of defects is high for CNT and reduces drastically for graphene (10-min growth time) (DOCX 2248 kb) [file 11671_2019_2935_MOESM1_ESM.docx]

*Additional file 1*

**Direct Growth of Graphene on Insulator Using Liquid Precursor via an Intermediate Nano Structured State Carbon Nanotube**

Pramoda K. Nayak*

Department of Physics, [Indian Institute of Technology Madras](http://aip.scitation.org/action/doSearch?field1=Affiliation&text1=Indian%20Institute%20of%20Technology%20Madras&field2=AllField&text2=&Ppub=&Ppub=&AfterYear=&BeforeYear=&access=), Chennai 600036, India

*E-mail: pnayak@iitm.ac.in

**
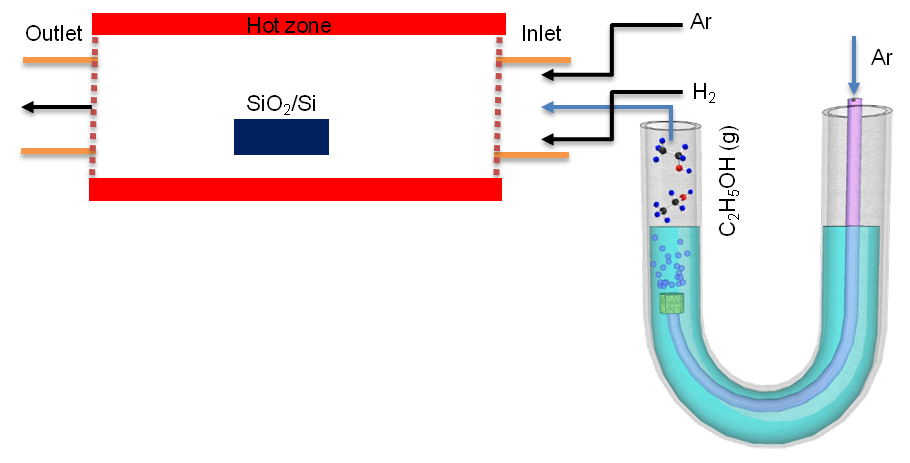
**

**Figure S1**. Schematic of the CVD growth of graphene from ethanol on SiO_2_/Si substrate.


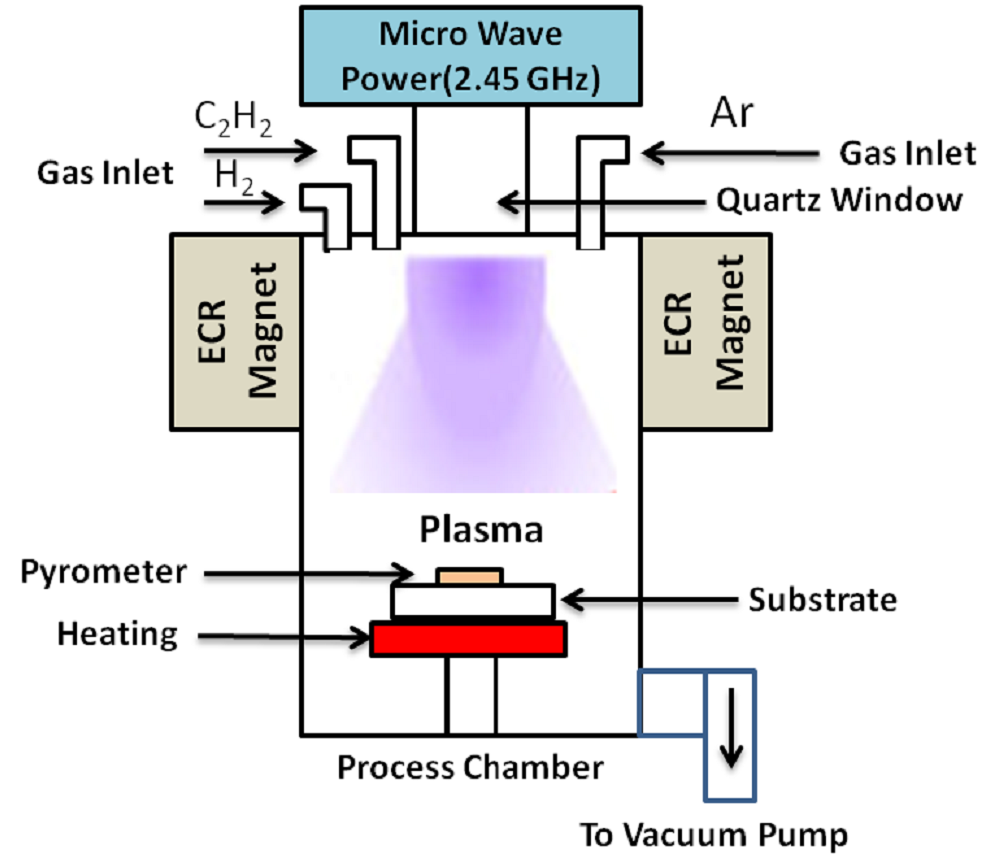


**Figure S2.** Schematic of ECR-CVD chamber.

**
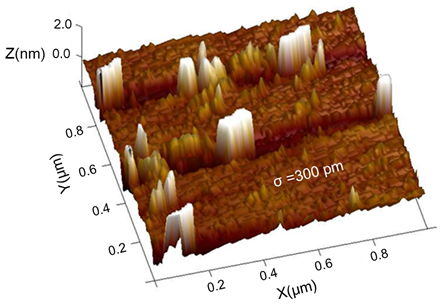
**

**Figure S3**. AFM image of 300nm SiO2/Si substrate showing step edges and surface roughness around 300 pm.

**
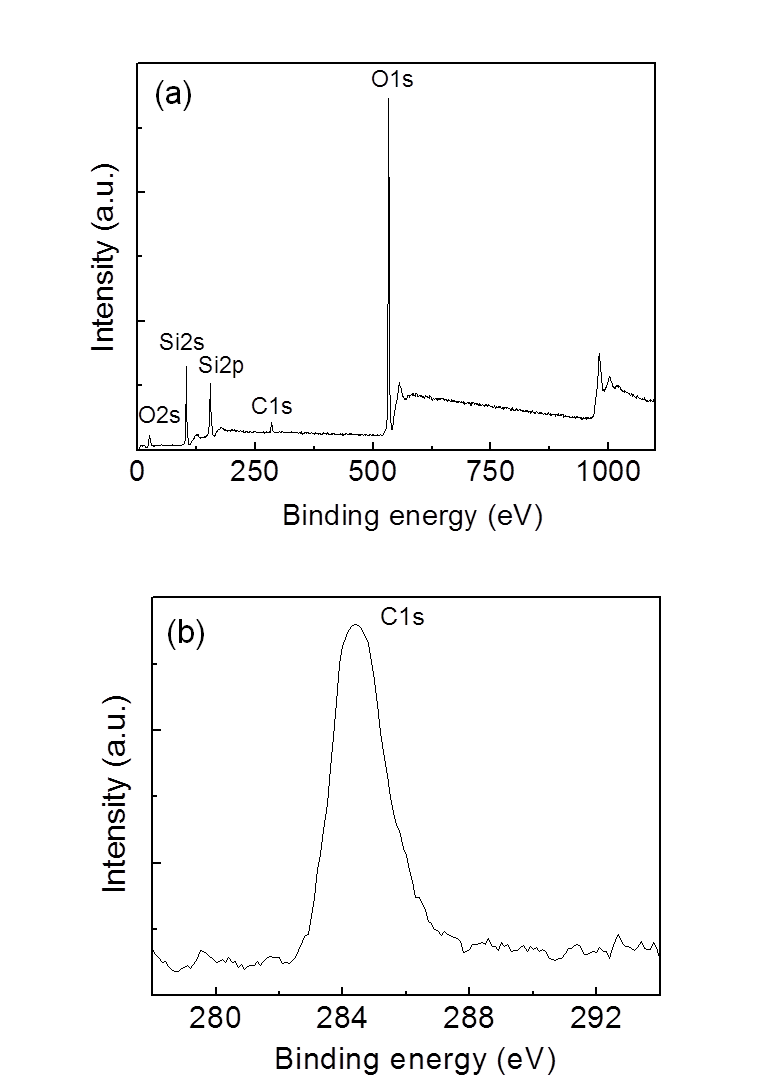
**

**Figure S4.** (a) XPS spectrum of the graphene film grown directly on SiO_2_ at 1100° C for 1 hour. (b) Evolution of C1s core-level spectrum of graphene grown on SiO_2_ for the same parameters. The narrow and symmetric intense peak at 284.4 eV assigned to the sp^2^-bonded C network.

**
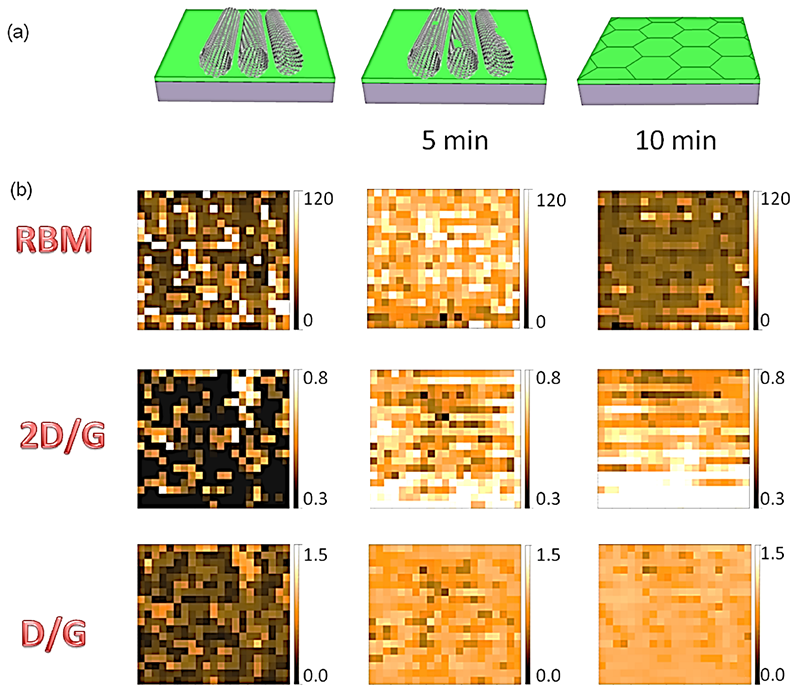
**

**Figure S5**. (a) Schematics of graphene growth on SiO_2_ using CNT as source material. (b) Raman intensity maps for RBM, 2D/G ratio and D/G ratio in 20×20μm^2^ regions of carbon nanostructures formed at different growth time 0, 5 and 10 min. The white rectangles in the Raman mapping correspond to RBM peak of CNT, which vanishes after 10 min growth time. The density of white rectangles (2D/G peak) is low for CNT and increases with increasing growth time. The density of black rectangles (D/G peak), which is the signature of defects is high for CNT and reduces drastically for graphene (10 min growth time).
